# Relationship between the extent of dissection and platelet activation in acute aortic dissection

**DOI:** 10.1186/s13019-015-0351-5

**Published:** 2015-11-10

**Authors:** Shu Zhang, Hong Qian, Qin Yang, Jia Hu, Changping Gan, Wei Meng

**Affiliations:** 1 Department of Emergency medicine, West China Hospital of Sichuan University, Chengdu, China; 2 Department of Cardiovascular Surgery, West China Hospital of Sichuan University, Chengdu, China; 3 Department of Radiology, West China Hospital of Sichuan University, Chengdu, China

**Keywords:** Acute aortic dissection, Inflammation, Platelet, Platelet activation, C-reactive protein

## Abstract

**Background:**

The extent of acute aortic dissection (AAD) was correlated with inflammation positively. On the other side, inflammation was negatively correlated with mean platelet volume (MPV), which can reflect platelet (PLT) activation. The aim of this study was to clarify the relationship between the extent of dissection and PLT activation.

**Methods:**

Between February 2010 and October 2013, 147 patients with acute aortic dissection (AAD) were divided into Group 1 (Stanford A, *n* = 59) and Group 2 (Stanford B, *n* = 88). Platelet count, MPV and platelet size distribution width (PDW) were measured to assess PLT activation. Additionally, the severity of inflammation was assessed via serum C-reactive protein (CRP), white blood cell (WBC) count and the neutrophil percent (Neut%). Computerized tomography (CT) was employed to analyze the extent of AAD. Volume tear index (VTI) was calculated as the false lumen (FL) volume divided by body surface area (BSA).

**Results:**

PLT count was significantly lower in group 1 than in group 2 (137.24 ± 31.04 × 10^9^/L vs 171.43 ± 27.57 × 10^9^/L, *P* < 0.001). The MPV/PLT ratio and PDW were significantly higher in the group 1 respectively(0.08 ± 0.02 vs 0.06 ± 0.02, *P* < 0.001; 22.65 ± 1.87 fl vs 20.69 ± 1.97 fl, *P* < 0.001). The CRP was significantly higher in group 1 than in group 2 (36.40 ± 8.89 mg/L vs 28.97 ± 8.48 mg/L, *P* < 0.001). VTI was significantly higher in group 1 than in group 2 (250.12 ± 27.82 vs 198.79 ± 24.52, *P* < 0.001). There was a significant negative correlation between VTI and PLT count (r = −0.673, *P* < 0.001), CRP and PLT count (r = −0.640, *P* < 0.001), respectively. There was a significant positive correlation between VTI and CRP (r = 0.670, *P* < 0.001), VTI and PDW (r = 0.601, *P* < 0.001), respectively.

**Conclusions:**

PLT activation and inflammation in AAD appear to be closely correlated with the extent of dissection, which possibly induced by the tear of aortic wall. Elimination of the false lumen is the goal of traditional surgery. Suppression of the PLT activation might be future targets of therapy in the prevention of systemic inflammation in AAD patients.

## Background

Mean platelet volume (MPV) and platelet size distribution width (PDW) are markers of platelet (PLT) size and variability, which can reflect PLT activation [1–3]. High grade inflammation is associated with low MPV whereas low grade inflammation is associated with high MPV; inflammation may play a role in pathogenesis of aneurysm formation and rupture [3]. Acute aortic dissection (AAD) is frequently accompanied by systemic inflammatory reaction, which is provoked by acute aortic injury and is reflected in an increment in serum C-reactive protein (CRP), white blood cell (WBC) count and pro-inflammatory cytokine levels [1–4]. In 2010, Manabu [5] reported extent of AAD was correlated with inflammation positively. We hypothesized that extent of AAD might correlate with the PLT activation. To our knowledge, these relationships have never been studied.

## Methods

### Patients

From February, 2010 to October, 2013. 341 consecutive AAD patients were admitted to the West China Hospital. The following categories of patients (*n* = 194 total) were excluded from our study, namely, those presenting later than 48 h from onset (*n* = 131), those with severe valve dysfunction (*n* = 27), those with recurrent AAD (*n* = 15), and those with malignant tumor or renal failure (*n* = 21). The remaining 147 AAD patients were enrolled in this study and examined prospectively. The patients were divided into 2 groups according to Stanford classification: Group 1 (Stanford A, *n* = 59) and Group 2 (Stanford B, *n* = 88). Blood samples were collected and axillary temperatures were recorded on the time of admission. Platelet count, PMV and PDW were measured to assess PLT activation. Serum CRP levels, WBC count and neutrophil percent (Neut%) were measured to assess the severity of inflammation. The study was approved by the medical ethics committee of West China Hospital. And all patients joined this study with informed consents.

### Analysis for extent of AAD

In each patient, contrast-enhanced helical CT was performed on a 64-row multidetector scanner (Brilliance 64, Philips Medical Systems, Cleveland, Ohio). Scans were obtained using 120 to 160 mL of a nonionic contrast agent continuously injected into the right antecubital vein at an infusion rate of 4 ml/s. To ensure maximum contrast intensity in the aorta, the region of interest (ROI) (threshold of 150 Hounsfield units) to trigger the beginning of the scan was placed in the ascending aorta. A second late arterial phase scan was performed, after a delay of 180 to 300 s from the end of the contrast scan, covering the same area [6]. The data obtained from CT scans were transferred to an Apple MacPro Quad 2.6 Ghz (Apple Corp, Cupertino, Calif) using digital imaging and communications in medicine (DICOM) data sets. The CT scans were stored and analyzed using the OsiriX software (Version 5.8.1 64-bit, Pixmeo SARL, Bernex, Switzerland).

**Fig. 1:**
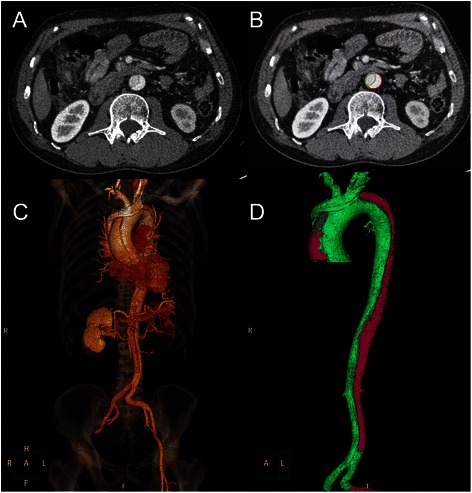
In each patient, we performed volumetric analysis of contrast-enhanced helical computed tomography (CT) covering the entire aorta starting from the innominate artery to the iliac bifurcation. Segmentations of the true lumen (TL, green) and false lumen (FL, red) were performed and volumes were calculated separately

Volumes were analyzed for the entire aorta from the innominate artery to the iliac bifurcation. The areas of interest, needed to calculate volumes, were drawn on CT scan manually (Fig. 1a and b, one selection every three scans), and the software provided automatically generating missing areas for every slice (Fig. 1c).

Additionally, the software allowed to perform automated segmentation (determining boundaries around a class of similar voxel intensity), which was used when appropriate and manually adjusted if necessary. The true lumen (TL) and false lumen (FL) volumes were calculated separately, and the overall volumes were calculated using the summation technique (Fig. 1d). Volumes computed by the software were expressed in cm^3^. For all patients, a volume tear index (VTI) was calculated as the FL volume divided by body surface area (BSA).

### Statistical analysis

Quantitative variables are presented as mean standard deviation and categorical variables as percentages. Continuous variables were compared using the Student’s *t*-test, Welch’s *t*-test or the Mann–Whitney *U*-test. Categorical data were compared using chi-square tests. Correlations between PLT activation, extent of AAD and inflammation parameters were analyzed by Pearson’s correlation coefficient. A *P* value < 0.05 was considered statistically significant. All data analysis was carried out using SPSS version 20.0 (SPSS, Inc, Chicago, IL, USA).

## Results

Table 1 showed the baseline characteristics, PLT indices, extent of dissection and inflammatory parameter. The lowest PLT count recorded in all patients was 84 × 10^9^/L. The highest VTI was 291.52. The highest CRP was 49.40 mg/L.

**Table 1:** Characteristics of the Patients

	Total	Group 1	Group 2	P Value
Baseline characteristics				
Age, years	47.13 ± 10.94	46.05 ± 11.40	47.85 ± 10.63	0.330
Male gender, (%)		48 (81.4 %)	65 (73.9 %)	0.291
BSA (m^2^)	1.69 ± 0.07	1.68 ± 0.07	1.69 ± 0.06	0.234
Time from onset of symptoms (hours)	24.29 ± 10.01	23.97 ± 9.80	24.51 ± 10.19	0.747
Hypertension		31 (52.5 %)	45 (51.1 %)	0.867
Diabetes		18 (30.5 %)	29 (33.0 %)	0.755
Dyslipidemia		13 (22.0 %)	23 (26.1 %)	0.571
Smoking		27 (45.8 %)	56 (63.6 %)	0.032
LVEF	52.62 ± 7.56 %	51.98 ± 7.55 %	53.05 ± 7.59 %	0.406
PLT indices				
PLT count (×10^9^/L)	157.71 ± 33.44	137.24 ± 31.04	171.43 ± 27.57	<0.001
MPV (fl)	10.56 ± 1.87	10.62 ± 1.90	10.51 ± 1.87	0.718
MPV/PLT ratio	0.07 ± 0.02	0.08 ± 0.02	0.06 ± 0.02	<0.001
PDW (fl)	21.48 ± 2.15	22.65 ± 1.87	20.69 ± 1.97	<0.001
Extent of dissection				
FL volume (cm^3^)	369.42 ± 57.12	419.31 ± 42.90	335.98 ± 37.81	<0.001
Volume tear index	219.39 ± 36.09	250.12 ± 27.82	198.79 ± 24.52	<0.001
Inflammation parameters				
CRP (mg/L)	31.95 ± 9.36	36.40 ± 8.89	28.97 ± 8.48	<0.001
WBC count	13.60 ± 2.95	14.00 ± 3.07	13.33 ± 2.85	0.177
Neut%	80.84 ± 5.18	81.83 ± 5.37	80.17 ± 4.96	0.056
Axillary temperature (C°)	37.79 ± 0.59	37.89 ± 0.60	37.72 ± 0.58	0.072

*BSA* body surface area, *LVEF* left ventricle ejection fraction, *FL* false lumen, *VTI* volume tear index, *CRP* C-reactive protein, *WBC* white blood cell, *Neut%* neutrophil percent

### Baseline characteristics

The mean age of all patients was 47.13 ± 10.94 years, and the majority of patients were male (76.9 %). Baseline characteristics were comparable between two groups, except that the smoking percentage in group 1 was lower than group 2 (45.8 % vs. 63.6 %, *P* = 0.032).

### PLT indices

The average of PLT count across all patients was 157.71 ± 33.44 × 10^9^/L. PLT count was significantly lower in group 1 than in group 2 (137.24 ± 31.0 × 10^9^/L vs 171.43 ± 27.57 × 10^9^/L, *P* < 0.001). The MPV/PLT ratio and PDW were significantly higher in the group 1 respectively (0.08 ± 0.02 vs 0.06 ± 0.02, *P* < 0.001; 22.65 fl ± 1.87 vs 20.69 ± 1.97 fl, *P* < 0.001). The MPV was no difference between two groups (*P* = 0.718).

### Extent of AAD

The average of FL volume of all patients was 369.42 ± 57.12 cm^3^ . FL volume was significantly larger in group 1 than in group 2 (419.31 ± 42.90 cm^3^ vs 335.98 ± 37.81 cm^3^, *P* < 0.001). VTI was significantly higher in group 1 than in group 2 (250.12 ± 27.82 vs 198.79 ± 24.52, *P* < 0.001).

### Inflammatory parameters

The CRP level was significantly higher in group 1 than in group 2 (36.40 ± 8.89 mg/L vs 28.97 ± 8.48 mg/L, *P* < 0.001). WBC count, Neut% and Axillary temperature were no difference between two groups.

### Correlation of PLT indices, extent of AAD and inflammation parameters

**Fig. 2:**
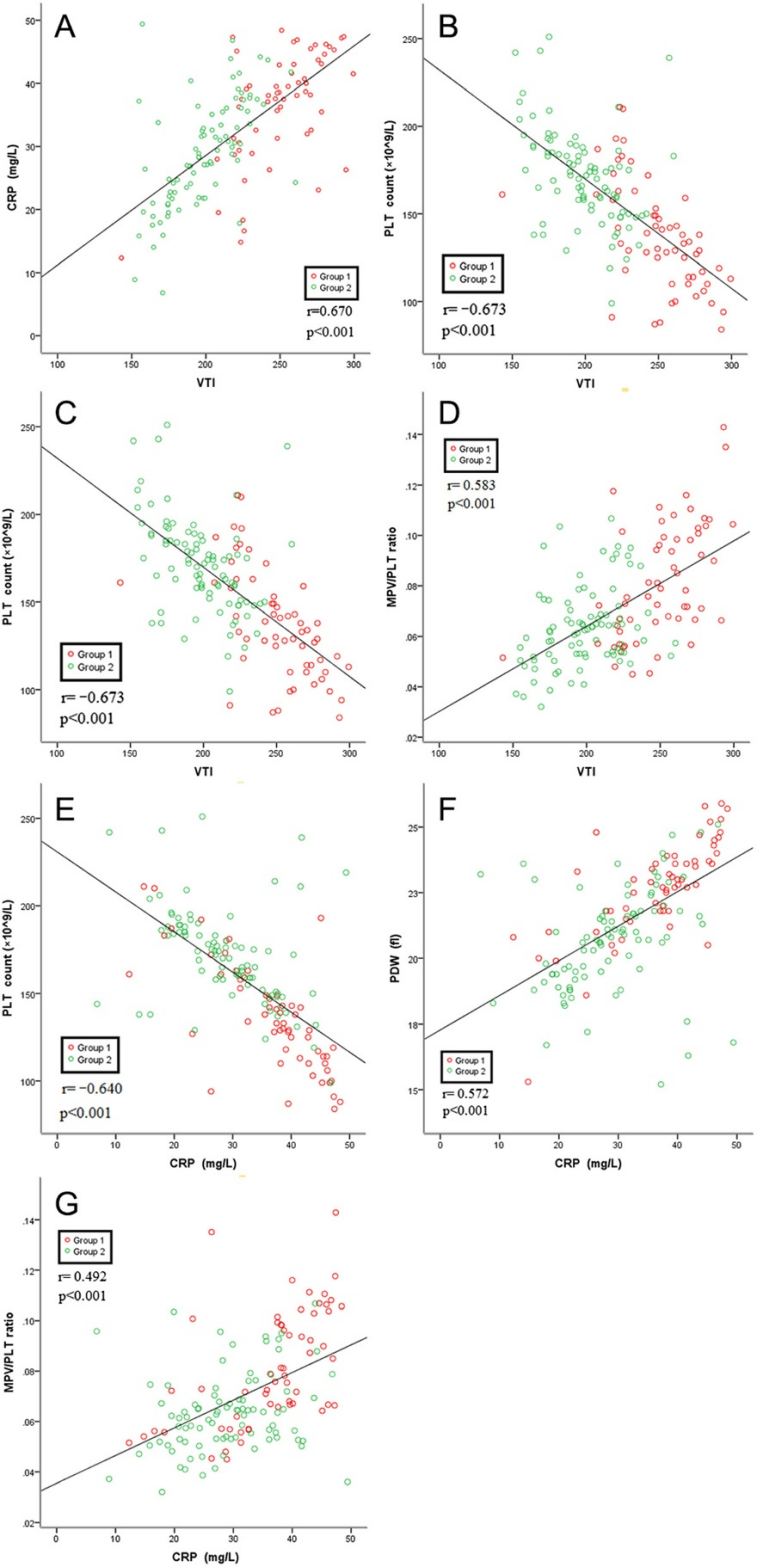
**a-g**: Correlation of PLT indices, extent of AAD and inflammation parameters

Correlation of PLT indices, extent of AAD and inflammation were presented in Fig. 2 There was a significant negative correlation between VTI and PLT count (r = −0.673, *P* < 0.001) (Fig. 2b), CRP and PLT count (r = −0.640, *P* < 0.001) (Fig. 2e), respectively. There was a significant positive correlation between VTI and CRP (r = 0.670, *P* < 0.001) (Fig. 2a), VTI and PDW (r = 0.601, *P* < 0.001) (Fig. 2c). The positive correlations were presented between VTI and MPV/PLT ratio, CRP and PDW, CRP and MPV/PLT ratio also. Although the correlations were not as strong as above two ((r = 0.583, r = 0.572, r = 0.492, *P* < 0.001, respectivly) (Fig. 2d, f, g).

## Discussion

Our study of AAD patients demonstrated greater extent of dissection in patients with a higher serum CRP (r = 0.670, *P* < 0.001). Patients with a greater extent of dissection also had lower PLT count (r = −0.673, *P* < 0.001);and higher MPV/PLT ratio and PDW respectively (r = 0.583, *P* < 0.001; r = 0.601, *P* < 0.001). Both CRP and PLT indices (PLT count, MPV/PLT ratio and PDW) were also significantly correlated. We believe our results provide strong evidence for the role of platelet activation in promoting inflammation in AAD. Sbarouni [7] found significant lower PLT count occurs in AAD patients also. In addition, the MPV/PLT ratio and the PDW are higher compared to chronic aortic aneurysms.

For aortic dissection, both Stanford and DeBakey classifications are qualitative, not quantitative [8]. In our study, we present a CT-based volume tear index to evaluate the extent of dissection. To best of our knowledge, there is no research report the relationship between extent of dissection and PLT activation in the world before. Diameter measurements are more widely used in most centers and clinical trials. Volumetric measurement is time-consuming and difficult to obtain in a clinic. However, evolution of postprocessing software that allows semi-automated measurements in a rather convenient and time-efficient fashion may, in the future, allows a more widespread use of this outcome parameters [6]. According to VTI, a new quantitative classification may be applied in the aortic dissection. 3D reconstruction-based volume analysis has been widely applied in many medical research fields, such as neurooncology, vascular intervention and so on [6, 9].

CRP, an acute phase reactant, is a sensitive and nonspecific inflammatory marker. It is produced mainly in liver by the stimulation of many cytokines, especially by IL-6. Its levels vary with inflammatory stages [10, 11]. Plasma CRP levels are time-dependent,which increased rapidly within 4–6 h, and reached peak values at 36–50 h, in acute inflammation, trauma or infectious diseases, and then decreased as the inflammation response was attenuated [12]. In accord with Kurabayashi’s research [5], our study showed the average CRP level was 6.39 times to normal value during the first 48 h. Our study showed there was significant correlation between inflammation and PLT activation. But the internal mechanism was not clear. Wu [13] reported that plasma platelet glycoprotein P- selectin and platelet cytolemma protein can adhere to circulating polynuclear neutrophils, aggravate the inflammation. And there was a significant positive correlation between VTI and CRP (r = 0.670, *P* < 0.001), Previous studies have reported that elevated plasma CRP levels were found in patients to predicting in-hospital clinical events of aortic dissection [11, 14]. So, whether VTI can be used as prognostic indicator in AAD patients, is worth further study. During the progress of AD, exposed collagen binds to von Willbrand Factors (vWF) and glycoproteins (GP) to activate platelets. PLT activation triggers multiple pathways: (1) release of α- and dense granules allows for additional feedback required for platelet activation and aggregation. (2) morphological changes in platelets occur, which results in production of pseudopod-like projections and tissue factor-rich microparticles [15].

## Conclusions

PLT activation and inflammation in AAD appear to be closely correlated with the extent of dissection, which possibly induced by the tear of aortic wall. Elimination of the false lumen is the goal of traditional surgery. Suppression of the PLT activation might be future targets of therapy in the prevention of systemic inflammation in AAD patients.
